# Immature defense mechanisms mediate the relationship between negative life events and depressive symptoms

**DOI:** 10.3389/fpsyt.2023.1341288

**Published:** 2024-01-11

**Authors:** Dandan Ma, Jinya Cao, Jing Wei, Jing Jiang

**Affiliations:** Department of Psychological Medicine, Peking Union Medical College Hospital, Chinese Academy of Medical Sciences and Peking Union Medical College, Beijing, China

**Keywords:** life events, defense mechanisms, depression, mediate, psychosomatic

## Abstract

**Objective:**

This study aimed to analyze the patterns of life events (LEs) and defense mechanisms in outpatients with depression and investigate the mediating role of defense mechanisms in the association between LEs and depressive symptoms in a psychosomatic outpatient sample in China.

**Materials and methods:**

All of 2,747 outpatients (aged 18–65) from psychosomatic department were investigated in this study. LEs, depressive symptoms, and defense mechanisms were assessed by the Life Events Scale (LES), Patient-Health-Questionnaire-9 (PHQ-9), and the Defense Style Questionnaire (DSQ), respectively.

**Results:**

Based on the optimal cut-off point of PHQ-9, 1840 (67.0%) patients had a PHQ-9 score of 10 or higher (depression group), and 907 (33.0%) had a score below 10 (non-depression group). The scores of Negative Life Events (NLEs), immature and intermediate defense mechanisms in the depression group were significantly higher than those in the non-depression group, while the scores of mature defense mechanisms were the opposite (*p* < 0.001). NLEs was directly related to depressive symptoms (*b* = 0.010, *p* < 0.001), and significant indirect effect via immature defense mechanisms (*b* = 0.008, *p* < 0.001) was observed.

**Conclusion:**

Immature defense mechanisms play an important mediating role in the relationship between NLEs and depressive symptoms. Helping patients improving defense mechanisms and dealing with NLEs may be of great help in the treatment of relevant patients.

## 1 Introduction

Depression is one of the most common psychiatric disorders characterized by persistent feelings of sadness, helplessness, hopelessness, reduced energy or fatigue, and considerable difficulty functioning in dealing with daily life (1). In the past decade, the prevalence of depression among young people has risen dramatically (2). Previous work has noticed the complexity of the etiology of depression (3, 4), and focusing solely on factors in a single field cannot understand the occurrence of depression. Today, it is a trend to understand depression from the perspective of biopsychosoical interactions (5, 6), and one most concerned environmental factors in this regard are life events (LEs) (7).

A severe independent event is sufficient to trigger depression (8). Studies have shown that LEs play an important role in the onset, remission, recurrence, persistence, and prognosis of depression (9, 10). LEs include negative life events (NLEs, e.g., bereavement; divorce) and positive life events (PLEs, e.g., marriage) (11). The impacts of NLEs on the development of depressive symptoms have consistently been documented (12). Mazure et al. reviewed previous case–control studies and found that depressed patients report higher rates of at least one severe NLEs than control group in both clinical and non-clinical samples (13). In addition, PLEs may also influence the depressive symptoms, yet there is no consensus. Shahar et al. (14) tested a 603 adolescent sample and found that PLEs are both protective and harmful factors. On the one hand, PLEs share an event-related aspect with NLEs, and on the other hand, PLEs can buffer the impact of NLEs on distress. Some studies have suggested that PLEs are related with lower depressive symptoms (15, 16), whereas others failed to show this relationship (17). According to diathesis-stress theories, vulnerable individuals more likely depressed when confront stressful LEs (18). There is a consensus that LEs are associated with depressive symptoms, but the underlying mechanisms between them has not been fully explored.

A growing body of literature has suggested that psychological factors play an important role in the onset of depression, depressed patients use more immature defense mechanisms (19). The concept of defense mechanism was first proposed by Freud in 1894 (20). Defense mechanism is defined as an automatic psychological process that can protect individuals from excessive anxiety and distress caused by internal or external dangers or stressors by distorting their perception of threatening events, thus maintaining psychological balance (21). Defense mechanisms can be grouped into three types: immature, intermediate, and mature defense mechanisms. Mature defense mechanisms indicate that individuals have a greater ability to adapt to reality and deal with social and emotional conflicts without distorting reality. Immature defense mechanisms indicate maladaptation to internal and external conflicts, which is related to severe psychopathology (22). Accumulating studies has confirmed that immature defense mechanisms predict greater severity of depression, poor treatment adherence, and unsatisfactory therapeutic outcomes (23, 24). Whereas, mature defense mechanisms are associated with less severity of depression and satisfactory treatment outcomes (25). Theoretically, the development of individual defenses is influenced by both biological and environmental factors (26). The relationship between defense mechanisms and LEs has also been explored. The increasing stressful LEs, the more use of immature defense mechanisms (27).

Further, previous studies suggested that immature defense mechanisms may play a mediate role in the relationship between childhood NLEs (such as childhood physical or psychological abuse) and psychopathological symptoms (28, 29). In the present study, we primarily tested the hypothesis that defensive mechanisms mediate the relationship between LEs and depressive symptoms. In addition, we also explored the characteristics of LEs and defensive mechanisms in outpatients with depression. We hope to provide more effective strategies for the treatment of patients with depressive symptoms.

## 2 Materials and methods

### 2.1 Participants

We recruited all the outpatients who attended the Department of Psychological Medicine of Peking Union Medical College Hospital from June, 2020 to April, 2023 and completed the following self-report questionnaires. The inclusion criteria were as follows: the age ranged from 18 to 65 years old, sufficient command of Chinese and cognitive capability to understand the self-report questionnaires. The exclusion criteria were adults with language barriers and unable to complete all the questionnaires. Anxiety disorders, depression disorders, obsessive-compulsive disorder, somatic symptom disorders, insomnia comprise the most common diagnosis in our outpatients. The project was approved by the Ethics Committees of Peking Union Medical College Hospital.

### 2.2 Measurements

#### 2.2.1 Depression

Depression was evaluated using the Patient-Health-Questionnaire-9 (PHQ-9) (30). PHQ-9 is a 4-point Likert scale used to describe the severity of depressive symptoms in the past two weeks. The total score of PHQ-9 ranging from 0 to 27, and the higher score indicates more severe depressive symptoms. PHQ-9 is an effective tool for monitoring patients with major depression in China, and the optimal cutoff point for detecting depression is 10 (31). In the present study, the Cronbach alpha value was 0.89.

#### 2.2.2 Life events

LEs were measured by the Life Events Scale (LES). LES is a self-report questionnaire consists of 48 items used to assess negative and positive LEs in terms of family life, work and social interactions. The purpose is to evaluate the nature and quantity of LEs to reflect perceived stress. Stimulation of a certain event = the severity of influence × the duration of influence × the number of occurrences. The total stimulus of LEs is the sum of the total stimulus of positive events and the total stimulus of negative events. Higher scores of the total stimulus of LEs represents greater stress perceived. LES has been widely used in China (32, 33). In the present study, the Cronbach alpha value was 0.82.

#### 2.2.3 Defense mechanism

Defense mechanism was assessed by the defense style questionnaire (DSQ) (34). DSQ includes 88 items, which can be divided into 4 factors. Factor I: immature defense mechanisms (e.g., projection, passive aggression); Factor II: mature defense mechanisms (e.g., sublimation, humor); Factor III: intermediate defense mechanisms (e.g., reaction formation, undoing); and Factor IV: concealment factor. It is a 9-point Likert scale, with ratings ranging from 1 (strongly disagree) to 9 (strongly agree) for each item. The closer to 9, the more frequently this defense mechanism is applied (35, 36). In the present study, the Cronbach alpha value was 0.91.

### 2.3 Data analysis

IBM SPSS Statistics 25.0 and PROCESS were used for statistical analysis.

Continuous variables and categorical variables were expressed as mean ± standard deviation (x ± s) and percentage, respectively. Based on the score of PHQ-9, all participants were divided into two groups. The comparison between the two groups was conducted using t-test for continuous variables and chi square test for categorical variables.

Characteristics with significant inter group differences (*p <* 0.05) were considered as potential predictive variables for depression and included in binary logistic regression using the ENTER procedure (total score of LES, score of negative LEs, score of family life, score of work, score of social interactions, score of immature defense mechanism, score of mature defense mechanism, and score of intermediate defense mechanism).

The correlation between NLEs and defense mechanisms was tested by the Pearson correlation coefficient.

Using the Bootstrap method with 5,000 samples, the mediating role of defense mechanisms (immature defense mechanisms, mature defense mechanisms and intermediate defense mechanisms) in the relationship between NLEs and depressive symptoms was tested through SPSS PROCESS (Model 4) (37). Gender (code: male = 1, female = 2), age and diagnosis (code: anxiety disorders = 1, depression disorders = 2, obsessive-compulsive disorders = 3, somatic symptom disorders = 4, insomnia = 5, and others = 6) were included as covariates to control for potential demographic impacts.

## 3 Results

### 3.1 Clinical features of patients with depression

In total, 2,747 outpatients were enrolled in the study. Based on the optimal cut-off point of PHQ-9, all participants were divided into two groups. Among them, 1840 (67.0%) patients had a PHQ-9 score of 10 or higher (depression group), and 907 (33.0%) had a score below 10 (non-depression group).

The age and percentage of male in the depression group were significantly lower than those in the non-depression group (*p* < 0.001).

The total LEs score of the depression group was significantly higher than that of the non-depression group (*p* < 0.001). Specifically, the scores of NLEs, family LEs, work related events and social related events in the depression group were significantly higher than those in the non-depression group (*p* < 0.001). However, no difference was found in the score of PLEs between the two groups (*p* = 0.181).

The scores of immature and intermediate defense mechanisms in the depression group were significantly higher than those in the non-depression group, while the scores of mature defense mechanisms were the opposite (*p* < 0.001) (see Supplementary Table S1 and Table 1).

**Table 1:** Clinical features of patients with depression.

	PHQ-9 ≥ 10 (*n* = 1840)	PHQ-9 < 10 (*n* = 907)	*χ*^2^/*F*	*p*
Female (%)	1,372 (74.6%)	613 (67.6%)	14.766	<0.001
Age (M ± SD)	33.71 ± 9.03	37.59 ± 10.02	14.144	<0.001
Total Life Events	160.48 ± 150.54	83.85 ± 95.47	130.050	<0.001
Negative Life Events	136.74 ± 137.89	61.98 ± 77.98	171.494	<0.001
Positive Life Events	23.73 ± 33.78	21.88 ± 34.95	0.485	0.181
Family	97.38 ± 109.37	55.40 ± 72.30	117.577	<0.001
Work	52.15 ± 56.91	23.50 ± 34.87	163.207	<0.001
Social	10.95 ± 22.24	4.95 ± 13.05	127.635	<0.001
Immature	4.69 ± 1.06	3.56 ± 0.93	16.362	<0.001
Mature	4.74 ± 1.14	5.11 ± 1.05	13.537	<0.001
Intermediate	4.54 ± 0.72	4.10 ± 0.73	1.024	<0.001

PHQ-9: Patient-Health-Questionnaire-9.

### 3.2 Predictor variables of depression

In binary logistic regression, NLEs, immature defense mechanisms, mature defense mechanisms and intermediate defense mechanisms were found to be predictor variables of a depression diagnosis. The explained variance was Nagelkerke *R*^2^ = 0.39 (see Table 2).

**Table 2:** Logistic regression analyses to predict depression.

Variables	β	SE	Wald	*p*	Exp (β)	95%CI
Total life events	−0.006	0.004	2.276	0.131	0.994	0.986–1.002
Negative life events	0.008	0.002	17.757	0.000^***^	1.008	1.004–1.012
Family aspect	0.003	0.004	0.574	0.449	1.003	0.996–1.010
Work aspect	0.008	0.004	3.621	0.057	1.008	1.000–1.016
Immature	0.934	0.080	138.081	0.000^***^	2.545	2.178–2.975
Mature	−0.348	0.053	42.435	0.000^***^	0.706	0.636–0.784
Intermediate	0.278	0.098	7.990	0.005	1.321	1.089–1.602

^***^p < 0.001. The statistically significant variables were output and listed by using ENTER procedures. Cox and Snell *R*^2^ = 0.277; Nagelkerke *R*^2^ = 0.385.

### 3.3 Correlations between NLEs and DSQ

Pearson correlation analysis showed that the score of NLEs was significantly and positively correlated with immature defense mechanisms (*r* = 0.363, *p* < 0.05), intermediate defense mechanisms (*r* = 0.187, *p* < 0.05), and negatively with mature defense mechanisms (*r* = −0.061, *p* < 0.05) (see Table 3).

**Table 3:** Correlations between negative life events and DSQ.

	Immature	Mature	Intermediate
Negative life events	0.363^**^	−0.061^**^	0.187^**^

DSQ: Defense style questionnaire. **p < 0.01.

### 3.4 The mediating role of defense mechanisms

The overall model accounted for a significant 40.2% of the variance in depressive symptoms, *F* (7, 2,739) = 262.680, *p* < 0.001, *R*^2^ = 0.402.

**Figure 1:**
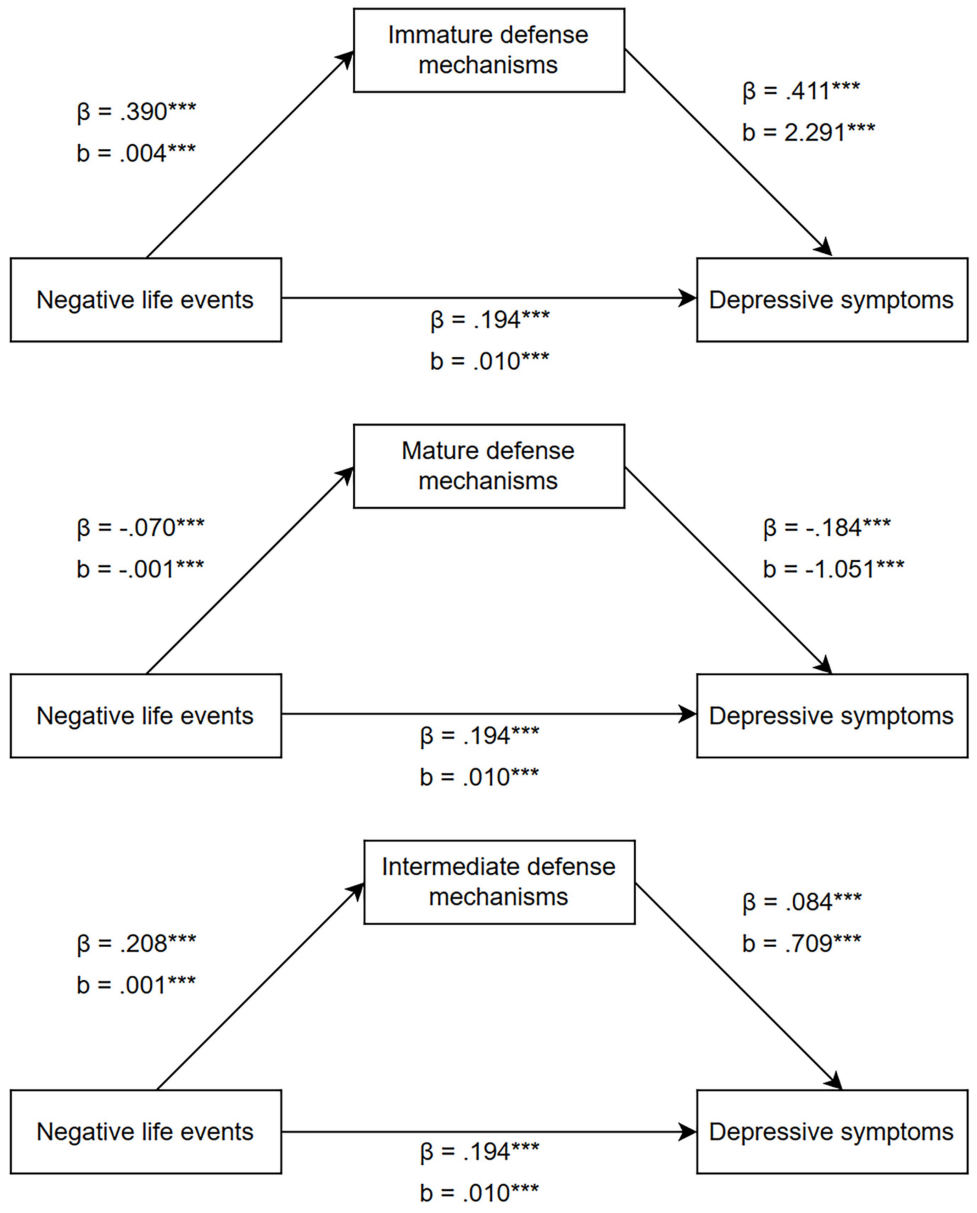
A structural equation model with direct and indirect effects between negative life events, defense mechanisms, and depressive symptoms. The effects of gender, age and diagnosis were controlled in the model. *β*: Standardized beta regression coefficient; *b*: Unstandardized beta regression coefficient. *** *p* < 0.001.

NLEs was directly related to depressive symptoms (*b* = 0.010, *p* < 0.001), and significant indirect effect via immature defense mechanisms (*b* = 0.008, *p* < 0.001), mature defense mechanisms (*b* = 0.001, *p* < 0.001) and intermediate defense mechanisms (*b* = 0.001, *p* < 0.001) were observed. The mediate effect of immature defense mechanisms was significantly higher than that of mature defense mechanisms and intermediate defense mechanisms. But there is no significant difference in mediating effect between mature defense mechanisms and intermediate defense mechanisms (see Figure 1 and Table 4).

**Table 4:** Unstandardized and standardized coefficients for all variables in the tested models.

Variable	*b* (95% CI)	SE	*β*	*t*	*p*
*Outcome variable: Factor Ι*					
Constant	4.997 (4.781, 5.213)	0.110	−	45.359	0.000^***^
NLEs	0.004 (0.003, 0.004)	0.000	0.390	22.883	0.000^***^
Gender	0.049 (−0.036, 0.135)	0.043	0.019	1.139	0.255
Age	−0.034 (−0.038, −0.030)	0.002	−0.286	−16.718	0.000^***^
Diagnosis	0.021 (−0.007, 0.049)	0.015	0.025	1.444	0.149
*Outcome variable: Factor II*					
Constant	4.498 (4.262, 4.735)	0.121	-	37.271	0.000^***^
NLEs	−0.001 (−0.001, 0)	0.000	−0.070	−3.642	0.000^***^
Gender	−0.016 (−0.109, 0.077)	0.048	−0.006	−0.336	0.737
Age	0.010 (0.006, 0.015)	0.002	0.089	4.657	0.000^***^
Diagnosis	0.049 (0.018, 0.081)	0.016	0.059	3.102	0.002
*Outcome variable: Factor III*					
Constant	4.912 (4.758, 5.067)	0.079	-	62.220	0.000^***^
NLEs	0.001 (0.001, 0.001)	0.000	0.208	11.198	0.000^***^
Gender	−0.135 (−0.196, −0.074)	0.031	−0.080	−4.348	0.000^***^
Age	−0.013 (−0.016, −0.010)	0.001	−0.162	−8.701	0.000^***^
Diagnosis	0.014 (−0.006, 0.034)	0.010	0.025	1.353	0.176
*Outcome variable: PHQ-9*					
Constant	4.722 (3.029, 6.416)	0.864	−	5.468	0.000^***^
NLEs	0.010 (0.008, 0.011)	0.001	0.194	11.941	0.000^***^
Factor Ι	2.291 (2.046, 2.535)	0.125	0.411	18.395	0.000^***^
Factor II	−1.051 (−1.239, −0.862)	0.096	−0.184	−10.942	0.000^***^
Factor III	0.709 (0.345, 1.074)	0.186	0.084	3.818	0.000^***^
Gender	0.988 (0.570, 1.406)	0.213	0.069	4.631	0.000^***^
Age	−0.070 (−0.091, −0.049)	0.011	−0.104	−6.625	0.000^***^
Diagnosis	0.040 (−0.099, 0.178)	0.071	0.008	0.558	0.577
*Direct effect of NLEs on PHQ-9*					
NLEs → PHQ-9	0.010 (0.008, 0.011)	0.001	0.194	11.941	0.000^***^
*Indirect effect of NLEs on PHQ-9*					
Total	0.010 (0.008, 0.011)	0.001	0.190	−	−
Factor Ι	0.008 (0.007, 0.009)	0.001	0.160	−	−
Factor II	0.001 (0.000, 0.001)	0.000	0.013	−	−
Factor III	0.001 (0.000, 0.001)	0.000	0.017	−	−
(C1)	0.007 (0.006, 0.009)	0.001	0.147	−	−
(C2)	0.007 (0.006, 0.009)	0.001	0.143	−	−
(C3)	0.000 (−0.001, 0.000)	0.000	−0.005	−	−

Factor Ι, Immature defense mechanisms; Factor II, Mature defense mechanisms; Factor III, Intermediate defense mechanisms. NLEs, Negative life events. PHQ-9, Patient-Health-Questionnaire-9. C1, Factor Ι minus Factor II; C2, Factor Ι minus Factor III; C3, Factor II minus Factor III. b, Unstandardized beta regression coefficient; β, Standardized beta regression coefficient; S.E., Standard Error; 95% CI: 95% Confidence interval. Gender coded: male = 1, female = 2. ^***^p < 0.001.

## 4 Discussion

In 2019, one paper published in JAMA had illustrated the ability of PHQ-9 to screen for and monitor depression, and the most common recommended positive case threshold was 10 points (38). Xiong et al. (31) found that the optimal cutoff point for detecting depression with PHQ-9 in the Chinese population is 10. In the present study, we divided all participants into two groups (depression group and non-depression group) according to the 10 score of PHQ-9. Our results showed that depression is more common among young women. This is consistent with the findings of an epidemiological study of DSM-5 major depressive disorder in adults in the United States (39).

### 4.1 LEs and depression

We observed that the LEs total scores and all LEs sub-scores (except for PLEs) were associated with depression. Logistic regression also showed that the NLEs score was the predictor of depression. Previous studies have consistently documented the impact of NLEs on the onset, remission, and recurrence of depression (16, 40, 41). Few studies have focused on the impact of PLEs on depressive symptoms, and the evidence is inconsistent (11, 16). One view has suggested that PLEs can directly or indirectly reduce depressive symptoms by buffering the effects of NLEs (42), while another view posits that PLEs themselves are also a source of stress (14, 43). In our present study, we did not find significant differences in PLEs between depressed and non-depressed patients. According to the content of the event, LEs can be divided into three dimensions: family life; work and study; social and other aspects. Similar to findings of other studies, we found all these three dimensions of LEs were related to depression (13, 44).

### 4.2 Defense mechanisms and depression

Our finding suggested that patients with depression are more likely to use immature and intermediate defense mechanisms, and less likely to use mature defense mechanisms, which is consistent with the case–control study by Peng et al. (45) conducted in populations with depression and non-suicidal self-injury. Accumulating literatures suggest that depressed patients mainly tend to use immature defense mechanisms (46, 47). As the symptoms of depression improve, patients show an increase in adaptive and a decrease in immature defenses (25). This suggest the influence of depression on defense mechanisms.

### 4.3 The mediate effect of defense mechanisms

Although the relationship between depression and NLEs has been established, the mediators of the link were not clear. Based on the above findings, we hypothesized that defense mechanisms may play a mediating role between NLEs and depressive symptoms. We found that immature defense mechanisms play a predominant mediating role. Similar to previous studies, Wang et al. (28) found that immature defense mechanisms had a significant mediate effect on the onset of bipolar disorder through childhood trauma in 86 patients with bipolar disorder and 43 healthy controls. In the study by Finzi-Dottan et al. (29) with 196 undergraduate students, immature defense mechanisms played a mediating role in the association between childhood emotional abuse and psychopathological symptoms. We also found the mediate effect of mature defense mechanisms and intermediate defense mechanisms in the relationship between NLEs and depressive symptoms, but significantly lower than that of immature defenses.

A study conducted by Araujo et al. (27) among 87 female adolescent patients found that immature defense mechanisms are related to life stressors. More sources of life stress are significantly positively correlated with an increase in the use of immature defense mechanisms. Accumulating evidence has converged to suggest that the increasing usage of immature defenses mechanisms may be associated with the severity of depression, increased risk of suicide attempts, reduced treatment compliance, and more treatment related adverse responses (48–50). Excessive use of immature defenses may cause serious damage to interpersonal and professional relationships, leading to severe psychological pain and worsening of psychopathological symptoms (25, 51). NLEs reflects perceived stressfulness, and the use of immature defense mechanisms reflects individual maladaptation.

From a psychodynamic perspective of view, defense mechanisms can be seen as part of ego function. Ego function has a basic level and can be influenced by other factors, such as psychopathology and stress, which can reduce ego function. But ego function can also mediate one’s ability to deal with stress and influence psychopathology (24). Thus, they have a bidirectional relationship with each other. In psychodynamic therapy, it is important to strengthen ego function in patients with a structural damage (a low basic level of ego function) and in patients with reduced ego function under stress and psychopathology.

In summary, we found that immature defense mechanisms and NLEs might be an important risk factor for depression. And defense mechanisms play a mediating role in the relationship between NLEs and depressive symptoms. These findings confirm the importance of NLEs and defense mechanisms in depression. Helping patients improving defense mechanisms and dealing with NLEs may be of great help in the treatment of relevant patients.

### 4.4 Limitations

The present study has the following limitations: (1) This study is a cross-sectional study and cannot reflect the causal effects between factors. (2) We used a self-assessment scale to screen patients with depression as a large-scale clinical study. (3) The control group was also patients from the department of psychological medicine, which may be influenced by confounding factors.

## 5 Conclusion

Immature defense mechanisms play an important mediating role in the relationship between NLEs and depressive symptoms. Helping patients improving defense mechanisms and dealing with NLEs may be of great help in the treatment of relevant patients.

## Data availability statement

The raw data supporting the conclusions of this article will be made available by the authors, without undue reservation.

## Ethics statement

The studies involving humans were approved by Peking Union Medical College Hospital (PUMCH). The studies were conducted in accordance with the local legislation and institutional requirements. The participants provided their written informed consent to participate in this study.

## Author contributions

DM: Writing – original draft. JC: Writing – review & editing. JW: Writing – review & editing. JJ: Writing – original draft.
