# Supplementary material for: Immature defense mechanisms mediate the relationship between negative life events and depressive symptoms
Source: Front Psychiatry. 2024 Jan 11;14:1341288. doi: 10.3389/fpsyt.2023.1341288 (PMC10808586; doi:10.3389/fpsyt.2023.1341288)
Supplement: Supplementary file 1 [file Table_1.DOCX]

**TABLE S1 The distribution of diagnoses among the sampled population (n = 2747).**

| Diagnoses | Participants, n (%) |
| --- | --- |
| anxiety disorders | 1256 (45.7%) |
| depression disorders | 1108 (40.3%) |
| obsessive-compulsive disorders | 76 (2.8%) |
| somatic symptom disorders | 72 (2.6%) |
| insomnia | 57 (2.1%) |
| others (eg. bipolar disorder, eating disorders) | 178 (6.5%) |
